# Multi-Omics Analysis of the Expression and Prognosis for FKBP Gene Family in Renal Cancer

**DOI:** 10.3389/fonc.2021.697534

**Published:** 2021-08-12

**Authors:** Zeqiang Sun, Xin Qin, Juanjuan Fang, Yueqing Tang, Yidong Fan

**Affiliations:** ^1^Department of Urology, Qilu Hospital, Cheeloo College of Medicine, Shandong University, Ji’nan, China; ^2^Department of Anesthesiology and Day Surgery, Dezhou People’s Hospital, Dezhou, China

**Keywords:** FK506-binding protein, renal cell carcinoma, prognostic model, diagnosis, biomarker

## Abstract

**Background:**

The FK506-binding protein (FKBP) is a family of intracellular receptors that can bind specifically to the immunosuppressant FK506 and rapamycin. Although FKBPs play crucial roles in biological processes and carcinogenesis, their prognostic value and molecular mechanism in clear cell renal cell carcinoma (ccRCC) remain unclear.

**Methods:**

Using pan-cancer data from The Cancer Genome Atlas (TCGA) and public databases, we analyzed the expression and correlation of FKBPs in 33 tumor types. Survival and Cox regression analyses were employed to explore the prognostic value of FKBPs. The relationship with tumor microenvironment and stemness indices was taken into account to evaluate the function of FKBPs. We constructed a risk score model to predict the prognosis of patients with ccRCC. The receiver operating characteristic (ROC) curve was performed to further test the prognostic ability of our model. Nomogram, joint effects analysis, and clinical relevance were performed to assist the clinician. Gene set enrichment analysis (GSEA) and cell line experiments were performed to investigate the function and molecular mechanisms of FKBPs in patients with ccRCC. Paired clinical specimens and multi-omics analysis were used to further validate and explore the factors affecting gene expression in ccRCC patients.

**Results:**

The expression levels of FKBP10 and FKBP11 were higher in ccRCC tissues than in normal tissues. The alteration in expression may be because of the degree of DNA methylation. Increased expression levels of FKBP10 and FKBP11 were associated with worse overall survival (OS). More importantly, GSEA revealed that FKBP10 is mainly involved in cell metabolism and autophagy, whereas FKBP11 is mainly associated with immune-related biological processes and autophagy. Cell Counting Kit 8 (CCK-8) and Transwell assays revealed that knockdown of FKBP10 and FKBP11 inhibits proliferation, migration, and invasion of the ccRCC cell line.

**Conclusion:**

FKBP10 and FKBP11 play important roles in ccRCC phenotypes and are potential prognostic markers as well as new therapeutic targets for patients with ccRCC.

## Introduction

There is increasing incidence of renal cancer diagnosis worldwide, and renal cancer is becoming a more prominent issue in our lives (1). Clear cell renal cell carcinoma (ccRCC) is the most frequent tumor type, accounting for 70% of all renal cancers diagnosed (2). Although targeted therapy and novel immunotherapeutic agents have been widely used, their efficacy is limited (3, 4). Because of the lack of effective methods for early diagnosis, patients with ccRCC generally have a poor prognosis (5). Therefore, valuable prognostic biomarkers are crucial for patients with ccRCC.

FK506-binding proteins (FKBPs), intracellular receptors that bind to FK506, are members of the immunophilin family (6–8). The FKBP gene family, comprising 16 members, can be modulated by various kinases and cellular factors. Activation of the FKBP gene family is associated with a range of biological activities, such as T-cell activation, cell metabolism, cellular homeostasis, tumor carcinogenesis, and tumor progression (6, 9). The majority of FKBP genes have peptidylprolyl cis/trans isomerase (PPIase) and tetratricopeptide repeat (TPR) domains within their protein structures (10). As the core domain, PPIase participates in cellular processes, such as transcription and protein formation (11, 12), and the TPR domain can bind to heat-shock protein 90 c(Hsp90) (13, 14). Increasing evidence suggests that FKBPs regulate cell cycle and survival and apoptotic signaling pathways and influence tumorigenesis and the response to chemotherapies and radiotherapies (6). FKBP1A binds to calcineurin and prevents NFAT dephosphorylation. T cells were activated to produce cytokines to regulate cell growth and proliferation *via* the PI3K pathway (6, 15–18). FKBP4 acts as a positive regulator of steroid receptors (19). In breast cancer models, especially in ER-negative breast cancer, high expression of FKBP4 can activate the PI3K–Akt–mTOR pathway to promote tumor growth and proliferation (20). FKBP5 is associated with androgen, glucocorticoid, estrogen, and mineralocorticoid receptors and acts as a negative regulator, except in the case of androgen receptors (21, 22). FKBP5, associated with BECN1, can also enhance autophagy to synergize with antidepressant action (23). FKBP5 not only influences physiological processes but also plays a key role in cancer development. In prostate cancer, FKBP5 can promote tumor carcinogenesis and progression by regulating androgen transcription (24). FKBP8 maintains cellular homeostasis *via* mediating mitophagy by interacting with LC3A and has been verified as an endogenous inhibitor of mTOR. The decrease in FKBP8 expression can induce apoptosis through the regulation of Bcl-2 (25–27). FKBP10 mediates aggressive phenotypes of stomach adenocarcinoma by regulating the PI3K signaling pathway (28). These studies demonstrate that FKBP genes play pivotal roles in tumorigenesis; however, there is currently no study of FKBPs in ccRCC, which means that the value of the FKBP family for predicting prognosis of ccRCC is still unclear and remains to be elucidated.

Genetics and epigenetics play major roles in the maintenance of cell identity and control of gene expression (29). Genetic changes can be divided into two major categories. First, single-nucleotide polymorphisms (SNPs) are the most common type of genetic change (30). Second, structural variation, including copy number variants (CNVs), refers to all base pairs that differ between individuals and that are not single-nucleotide variants (31). Recent studies have shown that genetic factors account for 35%–40% contribution of disease susceptibility (32, 33). SNPs in the FKBP5 gene, which cause the high expression of FKBP5, have been fundamentally linked to stress-related disorders, especially in psychiatric disorders (34, 35). Epigenetics affects gene expression without altering the DNA nucleotide sequence. Increasing evidence has shown that the aberrant epigenetic modifications in nucleic acids are associated with the occurrence of many diseases, including cancer, diabetes, Alzheimer’s disease, and many other age-related diseases (36). A recent study has shown that the different DNA methylation levels of FKBP5 are associated with varying responses to environmental influences and may play a role in how well people respond to psychological treatments (37).

With the development of large available databases and RNA sequencing techniques, the identification and application of new cancer biomarkers is becoming increasingly accurate and valuable. In this study, using pan-cancer analysis, we systematically analyzed the integral distribution, function, and prognosis of FKBP genes in humans. Using the data of patients with ccRCC from The Cancer Genome Atlas (TCGA) and Group on Earth Observations (GEO) database, we performed Cox regression, survival, and joint effects analysis and gene set enrichment analysis (GSEA) to screen the prognostic value and potential mechanism of the FKBP gene family in ccRCC. We constructed a risk score model and nomogram to assist clinicians with diagnostic and therapeutic decisions. Moreover, we preliminarily verified the effects of FKBPs on ccRCC proliferation and invasion, based on the cell phenotypes observed using an *in vitro* ccRCC model. Paired clinical specimens and multi-omics analysis were used to further investigate the impact factors of expression alteration.

## Materials and Methods

### Public Database Mining of FKBP Genes

The Genotype-Tissue Expression (GTEx) database (https://www.gtexportal.org) was used to analyze the distribution of the FKBP gene family in human normal organ tissues. The UALCAN database (http://ualcan.path.uab.edu/analysis.html) was used to perform survival and difference analysis. We used the Human Protein Atlas (HPA) database (https://www.proteinatlas.org/) to observe the protein expression of FKBPs. The Gene Expression Profiling Interactive Analysis (GEPIA2) database (http://gepia2.cancer-pku.cn) was used to draw survival maps.

### Data Handling

The RNA-seq data, DNA methylation data, copy number variation (CNV) data, somatic mutation data, and clinical information of pan-cancer analysis were downloaded from the TCGA (http://portal.gdc.cancer.gov/) and GEO (https://www.ncbi.nlm.nih.gov/geo/) database (TCGA-KIRC: The Cancer Genome Atlas Kidney Renal Clear Cell Carcinoma). The gene matrix of GSE40435 was downloaded from the GEO database. The Limma package from Bioconductor was used to perform difference analysis. Genes with an average count value >1 were excluded. *p* < 0.05 and |log2(FC)| > 1.0 was taken into account.

### Correlation Analysis and Construction of Prognostic Model and Nomogram

The R language corrplot package was used to evaluate correlations *via* Pearson correlation coefficient among FKBPs. Univariate and multiple stepwise Cox regression were performed by glmnet R packages. The risk score formula of the risk score model was as follows:

Risk score=α1∗E1+α2∗E2 …+αi∗Ei

In the formula, α represents the coefficient value. E represents the expression of FKBPs. The construction and evaluation of the nomogram used survival ROC and rms R packages. The screening standard was *p* ≤ 0.05.

### Gene Set Enrichment Analysis

C5 GO and C2 KEGG gene sets were used to perform GSEA analysis. The screening standard was *p* < 0.05, FDR < 0.05.

### Tumor Microenvironment Analysis

The stromal score and immune score were calculated by ESTIMATE analysis. R package “corrplot” was used to visualize the results.

### Stemness Indices Analysis

The data of stemness indices was downloaded from UCSC Xena (http://xena.ucsc.edu/). The Limma package and corrplot package were used to visualize the results.

### Cell Culture

786-O cells were obtained from the Cell Bank of the Chinese Academy of Sciences (Shanghai, China). Cell lines were routinely tested mycoplasma free and were authenticated by STR detection. The cell lines were cultured in RPMI1640 supplemented with 10% FBS.

### Cell Viability Assay

Cell proliferation was assessed by the Cell Counting Kit 8 (CCK-8) assay according to the manufacturer’s instruction (Dojindo Molecular Technologies, Rockville, MD). Briefly, 786-O (4 × 10^3^ cells/well) cells in 100 μl of medium were seeded in 96-well plates. After 12 h culture in 5% CO_2_ at 37°C in a humidified incubator, the medium was replaced by different concentrations of siRNA for 48 h. Afterwards, 10 μl of CCK-8 was added to each well. The cells were induced for another 1–4 h at 37°C according to the instructions of the manufacturer. Absorbance of each well was quantified at 450 nm by an enzyme-linked immunosorbent assay microplate reader (Tecan Trading AG, Switzerland).

### Invasion Assay

The *in vitro* invasive assay was performed using the Transwell system (24 wells, 8 mm pore size with polycarbonate membrane; Corning Costar, Lowell, MA, USA), whose upper chambers were coated with Matrigel (BD Biosciences, San Jose, CA, USA), forming a reconstituted basement membrane. Then, a total of 4 × 10^4^ 786-O cells, which were pre-transfected with 50 nM siRNA for 48 h, suspended in 100 μl of serum-free medium were seeded in the upper chambers, and 500 μl of medium containing 10% FBS was added to the lower chamber. After incubation for 24 h, the non-invaded cells in the upper chamber were gently removed with a cotton swab whereas the cells attaching to the lower surface were fixed in 4% paraformaldehyde for 15 min, followed by staining with 0.1% crystal violet for 20 min. The total number of cells invading and adhering to the lower surface was acquired in six representative fields using an Olympus light microscope.

### Migration Assay

The migration assay was performed in the same way as the invasion assay above except that the membrane was not coated with Matrigel. 786-O cells were pre-transfected with 50 nM siRNA for 48 h, and then 4 × 10^4^ 786-O cells were added to the upper chamber just as the invasive assay. After incubation for 24 h at 37°C, the cells were stained and counted in the same way as in the invasion studies.

### siRNA Transfection of Cells

About 2 × 10^5^ cells/well were seeded in six-well plates and transfected with small-interfering RNA (siRNA) (Santa Cruz, CA, USA) or a control siRNA using jetPRIME (polyplus-transfection, Illkirch, France).

### Real-Time Quantitative Polymerase Chain Reaction

Total RNAs were extracted using Trizol reagent according to the manufacturer’s specifications (TaKaRa Bio, Dalin, China). One microgram of total RNA from each sample was subjected to cDNA synthesis using the SuperScript III Reverse Transcriptase Kit (TaKaRa Bio, Dalin, China), for detection of the indicated genes and the housekeeping gene ACTIN. Each cDNA sample was amplified using SYBR Green (TaKaRa Bio, Dalin, China). The relative expression of the reported genes was determined using real-time PCR performed using an Applied Biosystems 7900HT Fast Real-Time PCR system. Fold change for every gene was calculated by the 2^−ΔΔCt^ method. Primers used for real-time PCR were as follows: Actin: F: 5’-TGACGTGGACATCCGCAAAG-3’; R: 5’-CTGGAAGGTGGACAGCGAGG-3′; FKBP10: F: 5’-GGCAGGGTTACATCATCCCC-3’; R: 5’-AAGATTAGCACGGCAGAGCC-3′; FKBP11; F: 5’-GTGTGTGGGAGAGAAGCGAA-3’; R: 5’-TGCAATCAGCTCCACGTCAT-3′.

### Statistical Analysis

Statistical analyses were performed using GraphPad Prism 8.0 and the R programming language version 3.5.2. Student’s *t*-test and one-way ANOVA were performed to determine significance. Each experiment was performed thrice, and *p*-values < 0.05 were considered significant.

## Results

### Pan-Cancer Analysis of FKBP Gene Family

**Figure 1:**
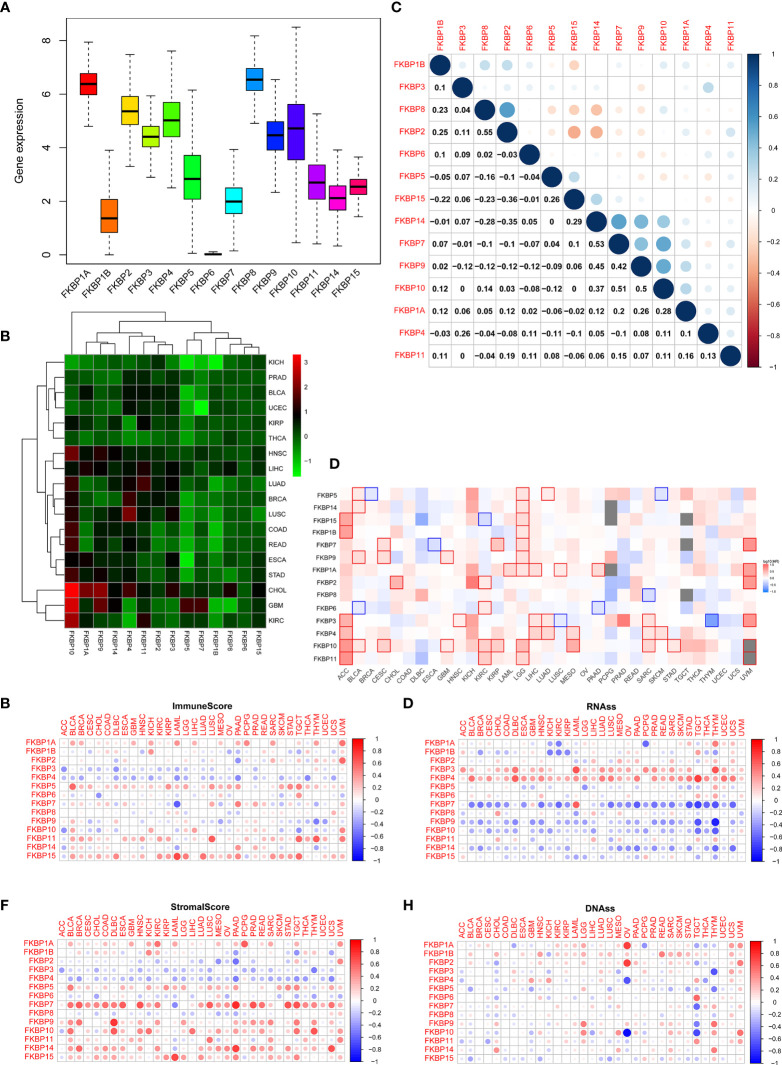
Pan-cancer analysis of the FKBP gene family. **(A)** The average expression level of the FKBP gene family among 33 tumor types in the TCGA database. **(B)** Differential expression analysis of the FKBP gene family in different tumor types. Red: upregulate. Green: downregulate. **(C)** Correlation analysis of the FKBP gene family by using Spearman correlation coefficient. **(D)** Survival analysis of the FKBP gene family in different tumor types. Red: positive correlation. Blue: negative correlation (KIRC: Kidney Renal Clear Cell Carcinoma). **(E, F)** The correlation of the FKBP gene family and tumor microenvironment. Red: positive correlation. Blue: negative correlation. **(G, H)** The correlation of the FKBP gene family and stemness indices. Red: positive correlation. Blue: negative correlation.

We performed a series of pan-cancer analyses using TCGA and public databases to explore the distribution, function, and prognosis of FKBP genes in humans. First, we analyzed the distribution of FKBP genes in human normal organ tissues in the GTEx database (**Supplementary Figure 1**). The results suggested that, compared with other organ tissues, the expression levels of FKBP1A-5, 9-11, 14, and 15 were above medium levels in human kidney tissues. FKBP6–8 showed lower expression levels. Second, by comparing human tumor tissues with corresponding normal organ tissues, we explored the expression level of FKBP genes in common tumor types using the UALCAN database (**Supplementary Figure 2**). Specifically, we found that FKBP1A, 5–11, and 15 were upregulated in kidney tumor tissues, whereas others were downregulated. Moreover, we evaluated the average expression level of the FKBP gene family and drew a heatmap to show the results of differential analysis using pan-cancer data from TCGA (**Figures 1A, B**). We found that the FKBP gene family had a higher overall expression level in all tumor samples except FKBP6. Most of members were downregulated in different cancer types except FKBP10. Moreover, using pan-cancer data from TCGA, we calculated the Pearson correlation coefficients of the FKBP genes (**Figure 1C**). Some genes showed strong correlations: FKBP2 and FKBP8 (*r* = 0.55, *p* < 0.05), FKBP7 and FKBP14 (*r* = 0.53, *p* < 0.05), FKBP7 and FKBP10 (*r* = 0.51, *p* < 0.05), and FKBP15 and FKBP2 (*r* = −0.36, *p* < 0.05). For further exploration, using the GEPIA2 database, we constructed survival maps between FKBP genes and common tumors using the Kaplan–Meier (KM) model (**Figure 1D**). The results revealed that FKBP2, 6, 10, and 11 were associated with the survival of patients with ccRCC. Above all, the expression and survival analysis suggested that FKBP2, 10, and 11 showed the most promise for further research. Increasing evidence has shown that cancer stem cells and the tumor microenvironment (TME) play a key role in the initiation and progression of tumors. We further validated the relationship between the FKBP gene family, cancer stem cells, and TME. The results showed that FKBP5, FKBP11, and FKBP15 were positively correlated with immune cells, whereas FKBP3 and FKBP4 were not (**Figure 1E**). Concurrently, FKBP7, FKBP9, and FKBP10 were positively correlated with stromal cells. Interestingly, FKBP3 and FKBP4 were negatively correlated with stromal cells (**Figure 1F**). The mRNA expression and DNA methylation data were used to calculate the stemness indices and their correlations with the FKBP gene family. The results suggested that the high expression of FKBP3 and FKBP4 contributed to stemness, and FKBP7, FKBP9, and FKBP10 had the opposite effects (**Figures 1G, H**).

### The Expression and Correlation Analysis of the FKBP Gene Family in ccRCC

**Figure 2:**
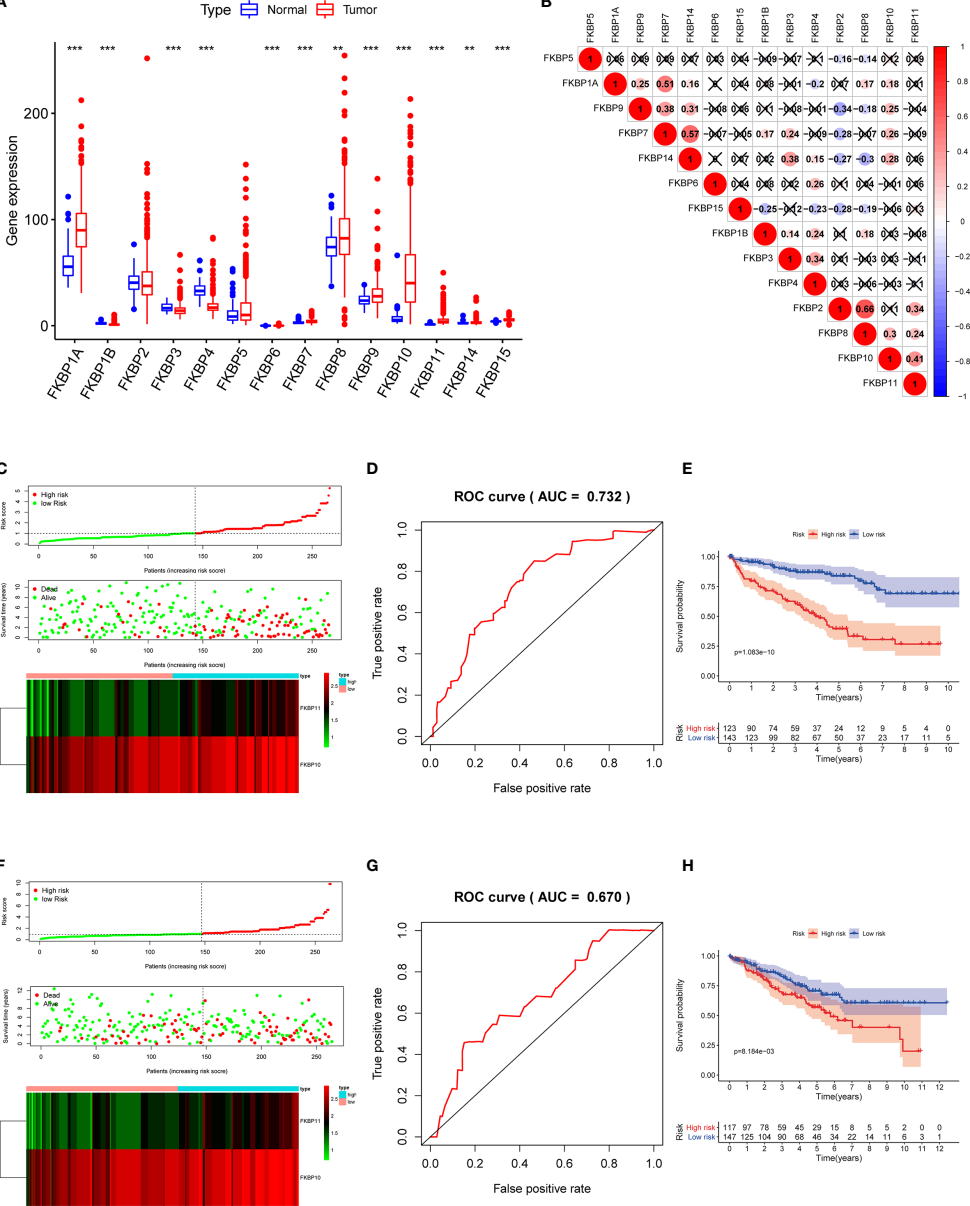
Expression of FKBP genes and risk score model construction based on the TCGA database with ccRCC patients. **(A)** Expression of FKBP genes between ccRCC tissues and normal kidney tissues based on the TCGA database. **(B)** Correlation analysis of the FKBP gene family in ccRCC by using Spearman correlation coefficient. **(C)** Risk score, expression heat map, and survival status in the training group. **(D)** ROC curves for forecasting OS in the training group. **(E)** Survival curve for low- and high-risk subgroups in the training group. **(F–H)** Similar analysis in the test group. *P < 0.05 **P <0.01 ***P < 0.001.

To precisely determine the expression and clinical significance of the FKBP gene family in ccRCC patients, we downloaded the data of ccRCC samples from the TCGA and GEO database (TCGA-KIRC: 72 normal samples and 539 tumor samples; GSE40435: 101 normal samples and 101 paired tumor samples). R software packages were used to normalize the data and perform a differential analysis. We found that the different expression levels of all FKBP genes reached statistical significance (**Figure 2A** and **Supplementary Figure S3A**). Among them, FKBP1A, 7, 10, 11, and 15 were remarkably upregulated, whereas FKBP1B and 4 were considerably downregulated. Based on the TCGA and GEO database, we calculated the Pearson correlation coefficients of FKBP genes (**Figure 2B** and **Supplementary Figure S3B**). The results suggested that FKBP2 and FKBP8 (*r* = 0.66, *p* < 0.05), FKBP7 and FKBP1A (*r* = 0.51, *p* < 0.05), FKBP7 and FKBP14 (*r* = 0.57, *p* < 0.05), and FKBP10 and FKBP11 (*r* = 0.41, *p* < 0.05) were markedly positively correlated, whereas the other genes showed a weak correlation.

### Prognosis-Related FKBP Gene Screening and the Construction of the Risk Model

To further investigate the role of FKBPs in the prognosis of ccRCC patients, we performed a univariate Cox regression analysis. The results revealed that only FKBP10 and FKBP11 were statistically significant (*p* < 0.05) (**Table 1**). Furthermore, we explored the effects of FKBP10 and FKBP11 on the overall survival (OS) and clinical outcomes of patients with ccRCC using multiple stepwise Cox regression analysis. The data in **Table 1** suggest that FKBP10 and FKBP11 were independent predictors in patients with ccRCC. We randomly divided the patients with ccRCC into a training group and a test group. In the training group, based on the results of multiple stepwise Cox regression analysis, we constructed a prognostic model that included risk score ranking, survival status, and heat maps of gene expressions (**Figure 2C**). The following formula was used to calculate the risk score of each patient:

Risk score=(0.6945∗ExpFKBP11)+(0.4383∗ExpFKBP10)

**Table 1:** Prognosis-related FKBP genes identified by Cox regression analysis.

	Univariate analysis			Multivariate analysis			
	HR	95% CI	*p*-value	coef	HR	95% CI	*p*-value
FKBP10	1.60	1.34–1.92	<0.001	0.44	1.55	1.22–1.96	<0.001
FKBP11	1.75	1.43–2.15	<0.001	0.69	2.00	1.47–2.72	<0.001

Receiver operating characteristic (ROC) and KM curves were used to evaluate our prognostic model. The area under the ROC curve (AUC) was 0.732, indicating a good prognostic significance. These results are shown in **Figure 2D**. Based on the median risk score, we divided approximately 267 patients with ccRCC into two subgroups and performed survival analysis. The results revealed that patients with high risks had a worse OS, whereas the low-risk subgroup showed improved outcomes (**Figure 2E**). We also used the data of the test group to perform the same analysis to evaluate our risk model. The results were comparable to the training group (**Figures 2F–H**), indicating that the prognostic model is reliable.

### Setup of a Predictive Nomogram According to FKBP10, FKBP11, and Clinical Features

**Figure 3:**
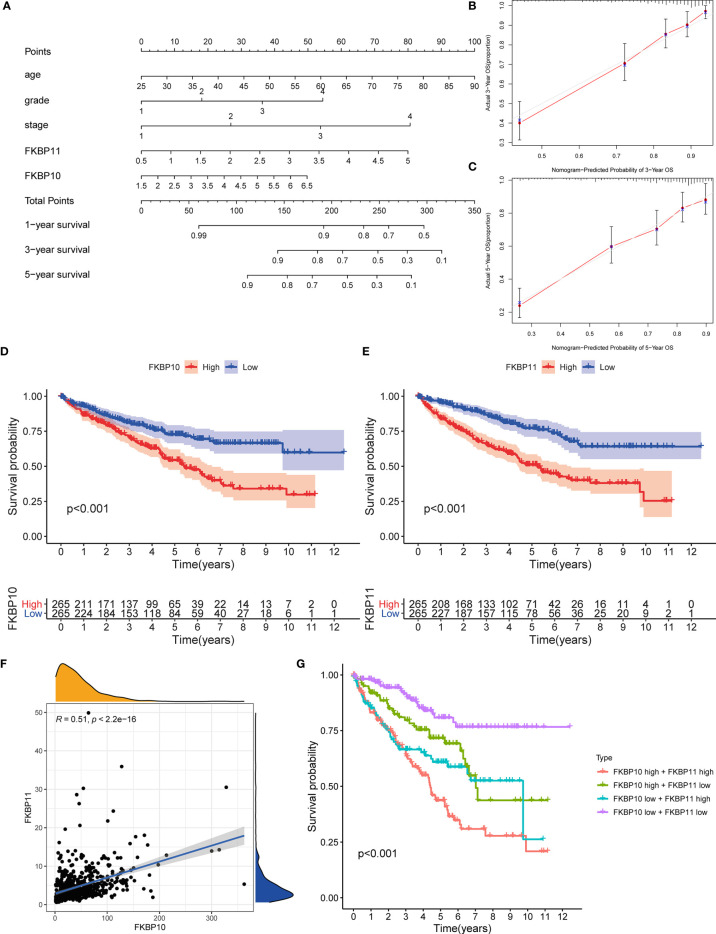
Nomogram construction and validation of the prognostic value of FKBP10 and FKBP11. **(A)** Nomogram for predicting OS of patients with RCC. **(B, C)** The calibration plot for the probability of survival at 3 and 5 years based on the TCGA database. **(D, E)** Survival analysis of FKBP10 and FKBP11 based on the TCGA database. **(F)** Correlation analysis by using the Spearman correlation coefficient. **(G)** Joint effects analysis of FKBP10 and FKBP11.

To further determine the relationship between our prognostic model and clinical features, univariate and multiple stepwise Cox analyses were performed (**Table 2**). The results revealed that risk score, age, tumor grade, and tumor stage are closely related to the survival time and clinical outcomes of patients with ccRCC and can be used as independent prognostic factors. Based on FKBP10 and FKBP11 expression and clinical features, we constructed a predictive nomogram (**Figure 3A**). By calculating and adding the score of each factor, we could predict the approximate survival rates of each patient and make clinical treatment decisions for ccRCC patients. The calibration plot for the probability of survival at 3 and 5 years showed promising prediction effects between nomogram and actual observations (**Figures 3B, C**).

**Table 2:** The prognostic value of different clinical features.

	Univariate analysis			Multivariate analysis		
	HR	95% CI	*p*-value	HR	95% CI	*p*-value
Age	1.03	1.01–1.05	0.003	1.03	1.01–1.05	0.013
Gender	0.75	0.49–1.15	0.185	0.79	0.50–1.25	0.312
Grade	2.38	1.77–3.20	<0.001	1.49	1.07–2.06	<0.001
Stage	1.89	1.58–2.26	<0.001	1.65	1.35–2.03	<0.001
Risk score	1.65	1.35–2.00	<0.001	1.17	0.91–1.49	<0.001

### Validation the Prognostic Value of FKBP10 and FKBP11 and Exploration of Molecular Mechanism

**Figure 4:**
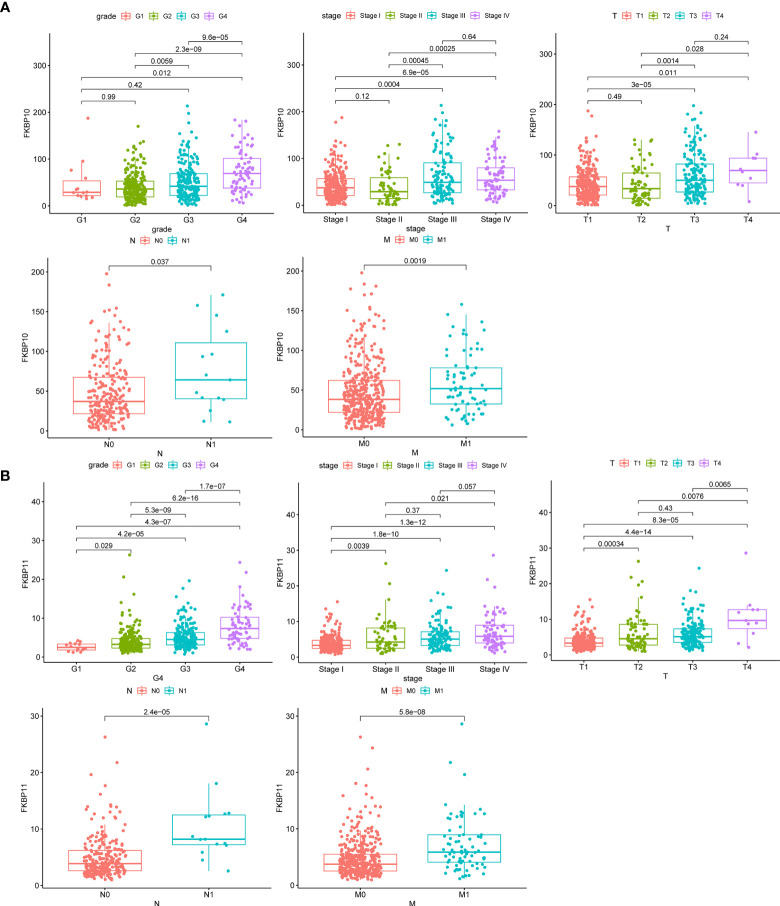
Clinical relevance of FKBP10 and FKBP11 in RCC. **(A)** The expression of FKBP10 in patients with RCC among the various pathologically differentiated grades and stages. **(B)** The expression of FKBP11 in patients with RCC among the various pathologically differentiated grades and stages.

**Figure 5:**
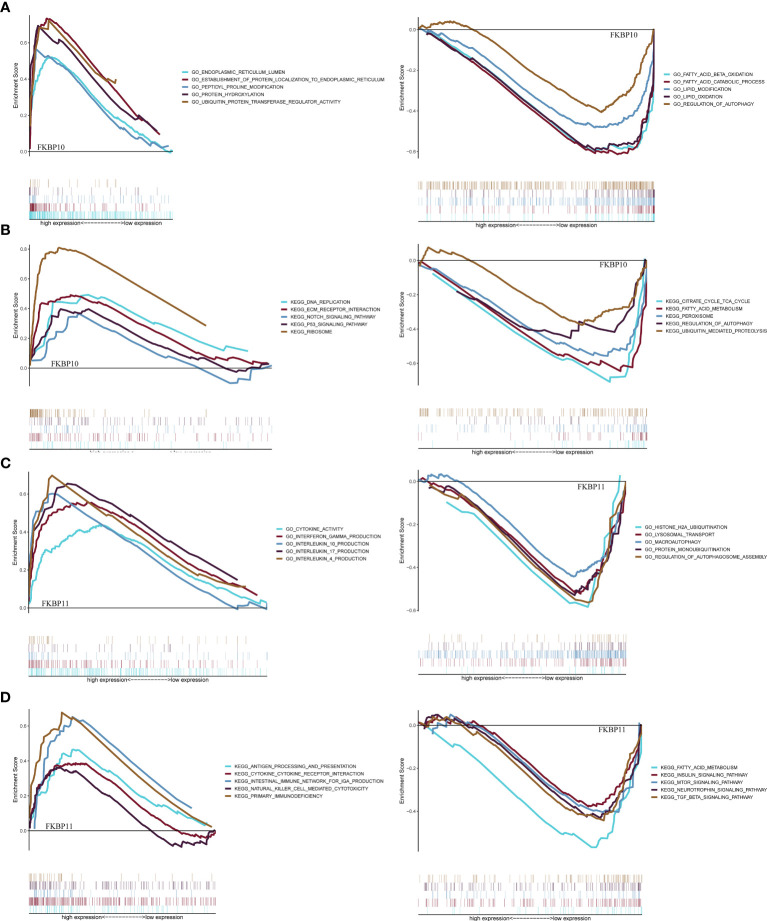
Gene set enrichment analysis and functional verification. **(A, C)** GSEA analysis of FKBP10 and FKBP11 with C5 GO gene sets. **(B, D)** GSEA analysis of FKBP10 and FKBP11 with C2 KEGG gene sets.

We further explored a more specific molecular mechanism and prognostic value of FKBP10 and FKBP11 in ccRCC. First, the Kaplan–Meier survival curves, using the log-rank test, were used to compare the relationship between FKBP genes and OS. The results showed that FKBP10 and FKBP11 are associated with the OS in patients with ccRCC (**Figures 3D, E**). The Spearman correlation coefficient was calculated as *R* = 0.51, *p* < 0.01 (**Figure 3F**). We also explored the joint effects of FKBP10 and FKBP11. The results showed that the group with low FKBP10 and low FKBP11 expression had the best outcome, whereas patients with high FKBP10 and FKBP11 expression had the worst prognosis (**Figure 3G**), suggesting that the difference in prognostic value for the combination of FKBP10-FKBP11 was more meaningful than any single marker. Second, we further analyzed the relationship between FKBP genes and clinical features (**Figures 4A, B**). The results revealed that FKBP10 and FKBP11 were closely related to the grade and stage of patients with ccRCC, suggesting that FKBP10 and FKBP11 had excellent prognostic value. Finally, we performed a single-gene GSEA. The GO enrichment analysis showed that high expression of FKBP10 is related to some biological processes and cellular component, such as endoplasmic reticulum lumen, establishment of protein localization to endoplasmic reticulum, lipid modification, and regulation of autophagy (**Figure 5A**). The KEGG enrichment analysis showed that high expression of FKBP10 is associated with the p53 signaling pathway, ribosome, peroxisome, and ubiquitin-mediated proteolysis (**Figure 5B**). Moreover, the GO enrichment analysis of the high expression of FKBP11 showed that FKBP11 is related to some biological processes, such as cytokine activity, interferon gamma production, lysosomal transport, and macroautophagy (**Figure 5C**). KEGG enrichment analysis showed that high expression of FKBP11 may take part in cytokine receptor interaction, fatty acid metabolism, and TGF beta signaling pathway (**Figure 5D**).

### Validation of Clinical Specimens and Multi-Omics Factor Analysis

**Figure 6:**
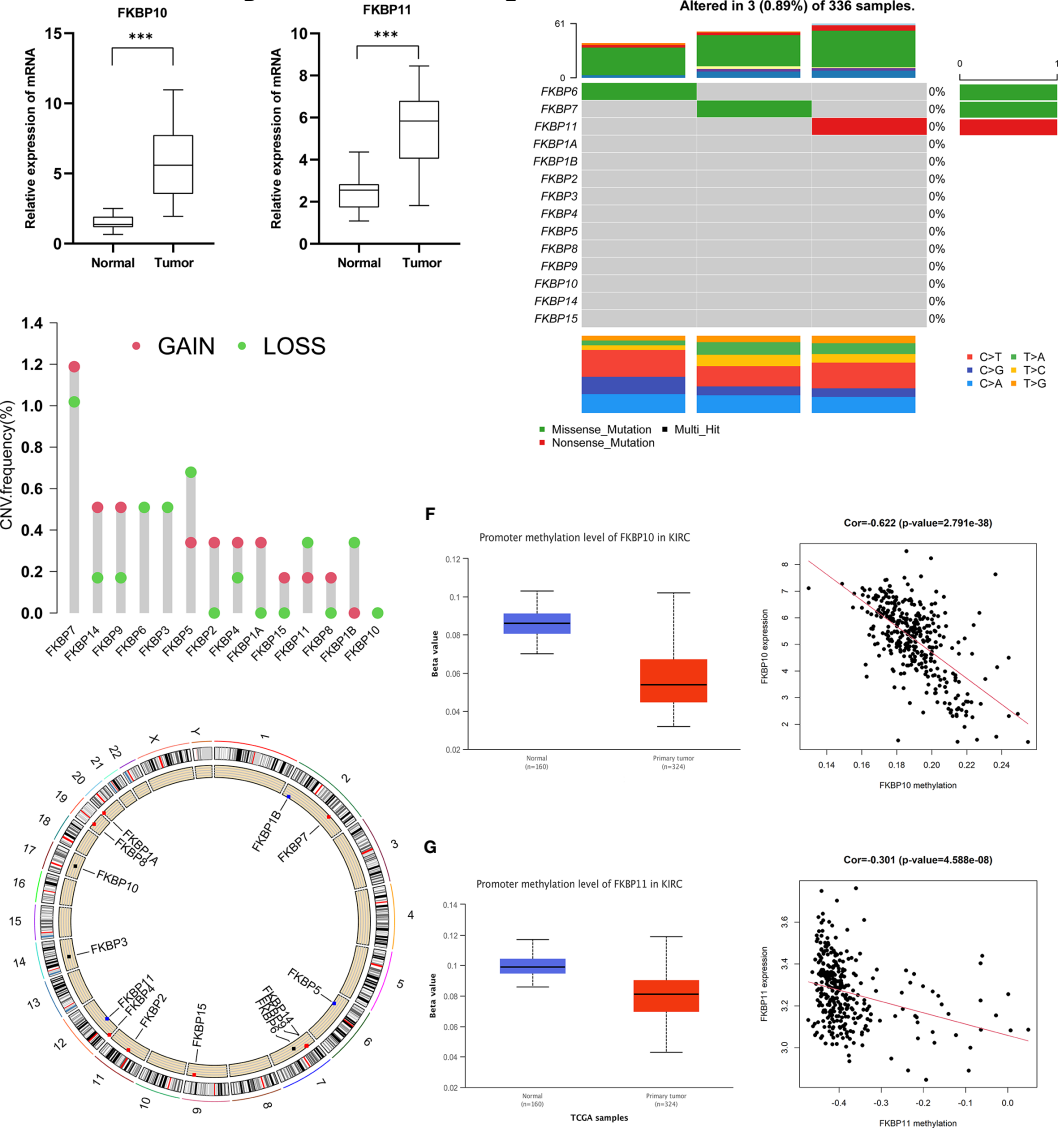
Validation of clinical specimens and multi-omics factor analysis. **(A, B)** Validation of the expression of FKBP10 and FKBP11 in 16 paired clinical specimens. **(C, D)** Copy number variation analysis of the FKBP gene family. **(E)** Somatic mutation analysis of the FKBP gene family. **(F, G)** The correlation of FKBP10 and FKBP11 expression and DNA methylation. *P < 0.05 **P < 0.01 ***P < 0.001.

To further verify our analysis, we downloaded the data of IHCs in ccRCC patients from the HPA public database (**Supplementary Figure S3C**). The differential protein expression of FKBP genes was similar to that of mRNA expression. We extracted RNA from 16 paired specimens of patients that were surgically treated for ccRCC in our hospital. We performed qRT-PCR to further validate the mRNA expression level. qRT-PCR results showed that the expression of FKBP10 and FKBP11 was increased in tumor tissue (**Figures 6A, B**). To further explore the reason for the high expression of FKBP10 and FKBP11, we performed multi-omics analysis. First, we considered the genetic alterations. By using the data of CNVs about ccRCC patients from the TCGA database, we found that the copy number of FKBP10 was barely affected, while the overall copy number alterations of FKBP11 were visible (**Figures 6C, D**). We also analyzed the frequency of somatic mutations in patients with ccRCC from the TCGA database. The results suggested that only one sample in each of FKBP11, FKBP6, and FKBP7 was mutated (**Figure 6E**). These data suggested that the high expression of FKBP10 and FKBP11 in ccRCC patients might not be because of genomic alterations. We then focused on epigenetic changes and analyzed the alterations of DNA methylation. The results showed that FKBP10 and FKBP11 have a low degree of methylation in tumor tissues. The expression and methylation showed moderate and weak correlation (**Figures 6F, G**). These results suggested that the alteration in expression might be due to epigenetic alterations. It provides one explanation of expression changes.

### Knockdown of FKBP10 and FKBP11 Inhibits Proliferation, Migration, and Invasion of the ccRCC Cell Line

We further investigated the effects of FKBP10 and FKBP11 expression on cell functions. We performed a CCK-8 assay to analyze cell viability and Transwell assay to test the motility ability of 786-O cells. Knockdown efficiency of FKBP10 and FKBP11 was examined using qRT-PCR (**Supplementary Figure 3D**). Knockdown of FKBP10 and FKBP11 significantly inhibited cell proliferation, migration, and invasion (**Supplementary Figures 3E, F**). The results of our experiments suggested that FKBP10 and FKBP11 might play important roles in the maintenance of the tumor characteristics of ccRCC.

## Discussion

The FKBP gene family is an intracellular receptor that can interact with other factors in cells by formation of a complex to regulate many signal pathways involved in many cellular event (6–9, 38). Many evidences have shown that FKBP dysregulation can be observed in various malignant tumors, indicating that they may play important roles in occurrence and development of cancers (39). However, the roles of FKBP in ccRCC are still unclear. In our study, we systematically analyzed the integral distribution, function, and prognosis of FKBP genes in humans by mining public database. We systematically analyzed the prognosis value of FKBPs and explored the underlying mechanism in ccRCC. Our data suggested that the high expression of FKBP10 and FKBP11 is associated with poor prognosis and can be independent predictors of ccRCC. Our results indicate that FKBP10 and FKBP11 can act as prognostic biomarkers for ccRCC.

Although many studies have explored the function and underlying mechanisms of FKBPs, the prognostic value of FKBPs has rarely been explored, especially in urinary system tumors. In our study, we first performed pan-cancer analysis to explore the integral distribution, function, and prognosis of FKBP genes in humans. We explored the differential expression of FKBPs in human normal organ tissues and some common tumors. We also explored the relationship between the expression of FKBPs and tumor grade, tumor stage, and survival state. The result revealed that FKBP2, FKBP10, and FKBP11 have prognostic value. Second, we analyzed ccRCC data from TCGA database to verify our hypothesis that FKBPs have prognostic values in patients with ccRCC. We analyzed different expression levels and screened prognosis-related FKBP genes. We found that only FKBP10 and FKBP11, but not FKBP2, had the most promise for further research. In addition, we constructed a risk score model and nomogram based on clinical features and the expression levels of FKBP10 and FKBP11 to help predict the prognosis of patients with ccRCC. Through verification experiments, our model and nomogram appeared to be significant and sensitive. FKBP10 and FKBP11 are independent prognostic indicators for ccRCC. Finally, we used paired clinical specimens and multi-omics analyses to further analyze the impact factors of expression alteration.

Additionally, we performed GSEA to further explore the specific molecular mechanism, of which FKBP10 and FKBP11 were involved. The results indicated that FKBP11 was related to immune-related biological processes and autophagy, such as interferon gamma production, lysosomal transport, macroautophagy, and TGF beta signaling pathway, whereas FKBP10 was involved in cell metabolism and autophagy. We explored the effects of FKBP10 and FKBP11 on ccRCC cell line *via* siRNA-mediated knockdown assays in an *in vitro* model. CCK8 and Transwell assays revealed that the knockdown of FKBP10 and FKBP11 inhibits proliferation, migration, and invasion of the ccRCC cell line.

However, our study has several limitations. Firstly, clinical information of ccRCC patients only came from the TCGA database and further verification is needed in other databases and clinical samples. Secondly, our results are only based on RNA sequencing and are not validated *via* other omics data platforms. Thirdly, less clinical information decreases the accuracy of our results. Finally, we have only demonstrated that FKBP10 and FKBP11 can affect the ccRCC phenotypes *in vitro*, and the underlying mechanisms need to be further studied in both *in vivo* and *in vitro* models.

## Conclusions

In summary, our study revealed the abnormal expressions and prognostic values of the FKBP gene family in ccRCC. We analyzed the relationship between FKBP expression and survival state in patients with ccRCC. Furthermore, we constructed a nomogram to predict the prognosis of patients with ccRCC. Additionally, we performed GESA analysis and *in vitro* experiments involving the ccRCC cell line to determine the function and underlying mechanism of FKBPs. All these results suggest that FKBP10 and FKBP11 are potential prognostic markers and novel therapeutic targets for patients with ccRCC.

## Data Availability Statement

The datasets presented in this study can be found in online repositories. The names of the repository/repositories and accession number(s) can be found in the article/**Supplementary Material**.

## Ethics Statement

The studies involving human participants were reviewed and approved by Ethics Committee of the Shandong University Qilu Hospital. The patients/participants provided their written informed consent to participate in this study.

## Author Contributions

ZS and XQ analyzed the data. JF finished the validation of clinical specimens and cell experiments. YT and YF designed the project, selected the analyzed results, and wrote the paper. All authors contributed to the article and approved the submitted version.

## Funding

This work was supported by the National Natural Science Foundation of China (No. 81672522), the Department of Science and Technology of Ji’nan city (No. 201805030), the China Postdoctoral Science Foundation Grant (2019M650164), and the Shandong University and Karolinska Institute Cooperative Research Project (SDU-KI-2020-11).

## Conflict of Interest

The authors declare that the research was conducted in the absence of any commercial or financial relationships that could be construed as a potential conflict of interest.

## Publisher’s Note

All claims expressed in this article are solely those of the authors and do not necessarily represent those of their affiliated organizations, or those of the publisher, the editors and the reviewers. Any product that may be evaluated in this article, or claim that may be made by its manufacturer, is not guaranteed or endorsed by the publisher.
