# Supplementary material for: Multi-Omics Analysis of the Expression and Prognosis for FKBP Gene Family in Renal Cancer
Source: Front Oncol. 2021 Aug 12;11:697534. doi: 10.3389/fonc.2021.697534 (PMC8406630; doi:10.3389/fonc.2021.697534)
Supplement: Supplementary file 1 [file DataSheet_1.docx]

Supplementary Material

# Supplementary Figures and Tables

## Supplementary Figures


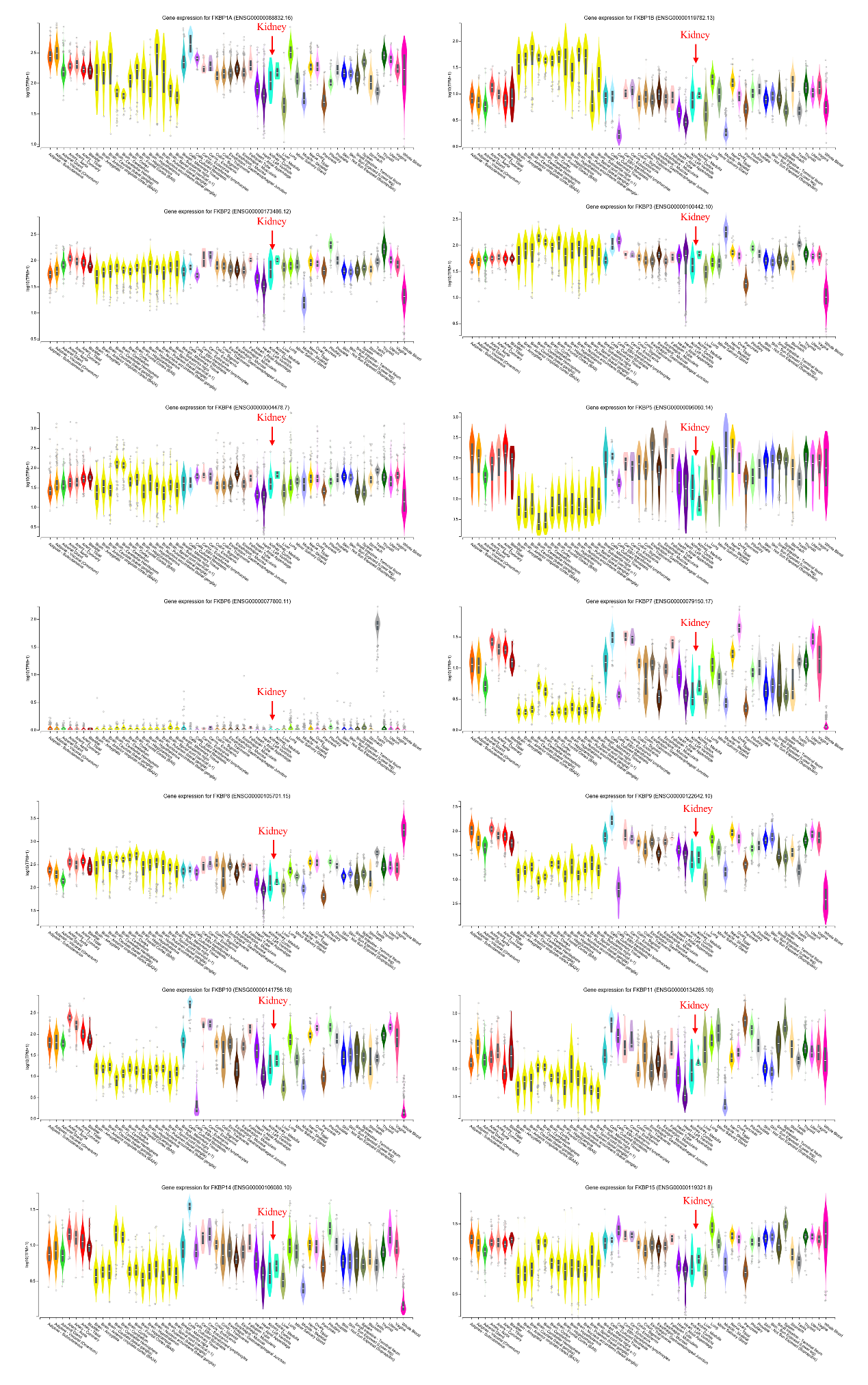


**Supplementary Figure 1.** Expression distribution of FKBP genes in human normal organ tissues using GTEx database.

c
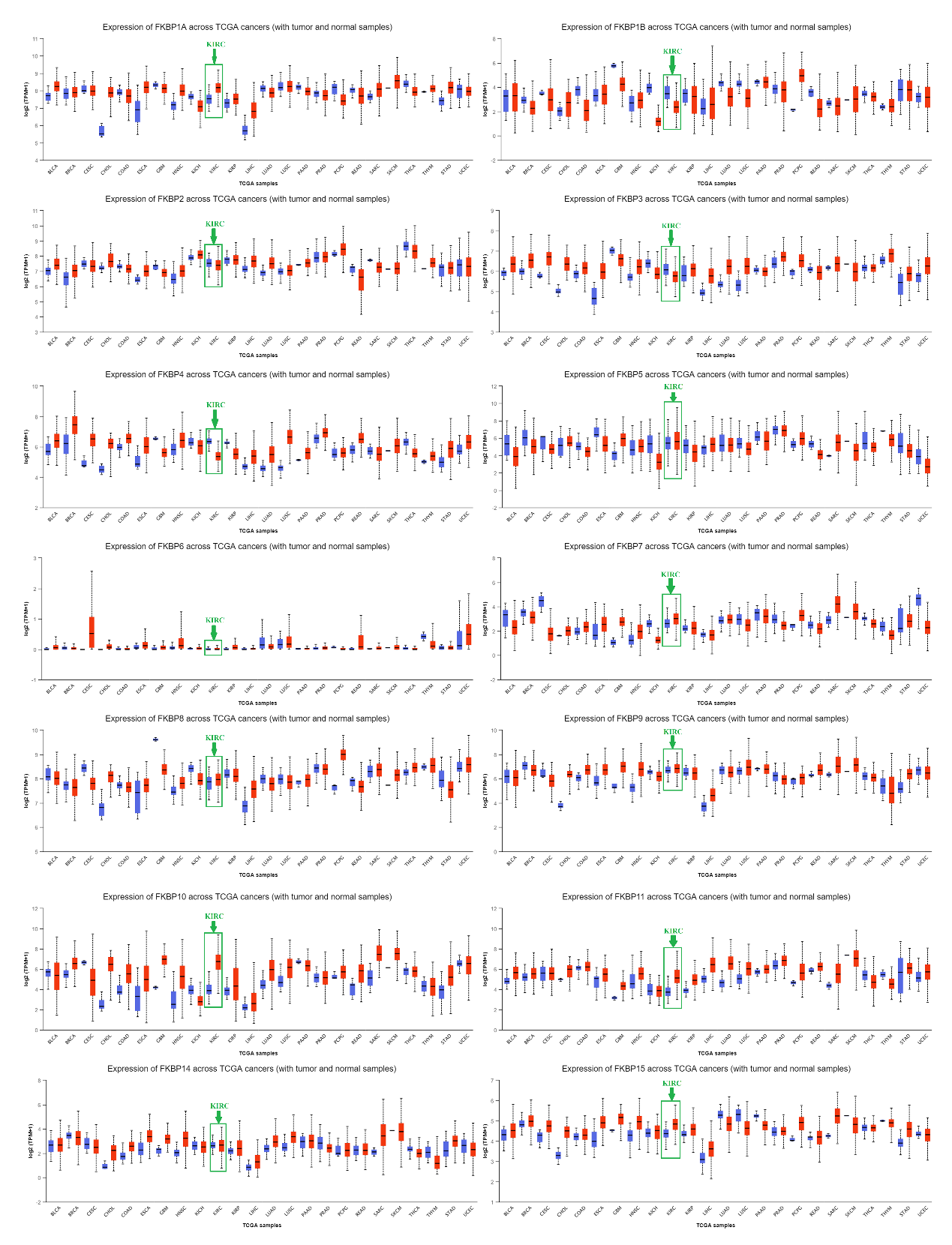


**Supplementary Figure 2.** The different mRNA expression of FKBP genes in 24 kinds of common tumors and corresponding normal tissues using Ualcan database.


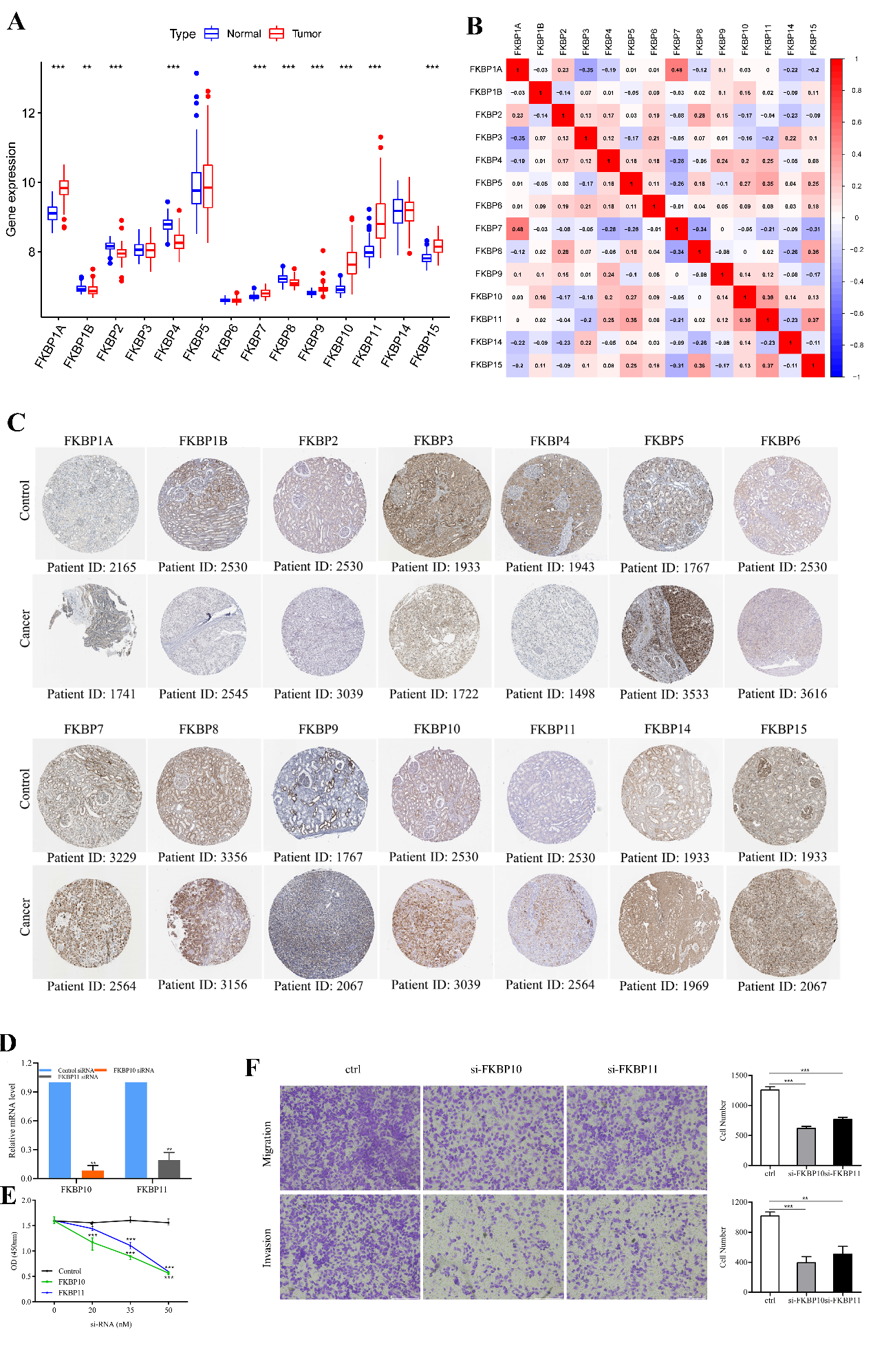


**Supplementary Figure 3.** (A) Expression of FKBP genes between ccRCC tissues and normal kidney tissues based on GEO database (GSE40435); (B) Correlation analysis of FKBP gene family in ccRCC by using spearman correlation coefficient based on GEO database (GSE40435); (C) The protein expression of FKBP genes in RCC by HPA database; (D) The examination of knockdown efficiency of FKBP10 and FKBP11 by qRT-PCR; (E) Cell viability analysis in 786-O cell line; (F) Transwell analysis in 786-O cell line.
